# Unusual Action of Anæsthetics

**Published:** 1878-02-20

**Authors:** 


### UNUSUAL ACTION OF ANÆSTHETICS.

Dr. Keyser, in the *Medical and Surgical Reporter*, remarks:

During the course of a number of years study in Europe, I was present and assisted in the administration of chloroform very many times, in the various clinics and hospitals; and since my return home to this city I have had considerable experience with chloroform, ether, and bichloride of methylene, as anæsthetics, in my hospital clinics, as well as in private practice; the following cases have come under my observation, all of which I have no doubt can be authenticated by the experience of others as soon as the interest in compiling such cases is aroused.

In relating the cases I will present, first, those of peculiar hallucinations; second, as to the retention of consciousness during the operation without feeling pain; third, as to anæsthesia while asleep, without awakening.

While I was attending Prof. von Graef's clinic, in Berlin, a young lady of about 19 years came in, for the operation of neurotomy of the supra-orbital nerve. It was necessary to anæsthetize her, chloroform being used. During which act, in the presence of the class, she passed through all the actions and emotions of coition. Dr. von Graefe called attention to this, as one of the effects, at times of inhaling chloroform, and advised the necessity of having several present when chloroform was administered to the opposite sex.

In my clinic in the Wills Eye Hospital, in 1874, a female of seventeen years of age came to me to be operated on for strabismus convergens. She wished to be etherized, so as to prevent her feeling any pain. During the administration of the anæthetic by the house surgeon, in the presence of several visiting students and physicians, as well as the regular clinic assistants, she, after a few minutes' inhalation, began talking in the most lascivious manner, accompanied with all the actions and emotions of sexual intercourse; and when coming out from under the deep anæsthesia, after the operation, the same hallucination and actions possessed her. When free from its influence she claimed that one of the assistants had had sexual intercourse with her,

This idea was given up, however, after being assured that such was not the case, and of the number present at the operation. Some one must have mentioned the whole or part of the affair to her, for on her appearance at the next clinic day she very modestly apologized to me for her actions, saying, "that she could not help it, as she knew nothing at the time."

In the spring of 1876, I was obliged to etherize a young lady, so that I could make the operation of tearing loose posterior synechia, and during the inhalation, which was administered by my friend, Dr. Frank Fisher, her eyes became uncovered for a moment, in which time she opened them and looked staringly and frightened at me, as I sat by her side holding the pulse. She struggled to get away, and attempted to scream. Her eyes were at once covered, and the ether pushed to complete anæsthesia. After coming out from under its influence, she told the young lady friend with whom she was staying, and who was present at the operation, that "I had turned into a wild cat; she was positive of it." For ten days she was so impressed with this delusion that she would not look at me, and it was with difficulty that I could treat the eye.

In relation to the second question, "Can there be retention of consciousness during the operation, without feeling pain?" the following two cases will show that such can be.

In the summer of 1863, while I was assisting Dr. Hensey in the surgical wards of the Citizen's Hospital (Burgerpital,) in Berne, Switzerland, a case of amputation of the leg at the lower third of the femur was made on a gentleman of that city, in which it was necessary to administer an anæsthetic. After inhaling chloroform for a few minutes he lost all feeling, but continued to converse with Dr. Hensey, and told him to go on with the operation, but before doing so prop him up, so that he could see the whole affair. He looked at the operation very quietly, and denied feeling the least pain, nor did he show in the least that he experienced any.

In April, 1871, in my clinic in the Philadelphia Eye and Ear Infirmary, I made an operation for shrinkage of a staphylomatous eyeball, on a lady of thirty-five years of age, who, after inhaling a mixture of chloroform and ether a few minutes, lost all feeling, but not consciousness, and the operation was made without the least sensation of pain, as assured me by the patient, who remained perfectly quiet, and answered promptly every question that I put to her. The anæsthetic was administered by my assistants, Drs. J. W. Millick, D. C. Lloyd and W. F. Church, in the presence of several students who were attending my clinic and lectures at the time.

To the third question, "Can one be anæsthetized during sleep without awakening?" the following will show:

July 26th, 1876, I was called to Lebanon, Pa., to enucleate the eyeball of a boy, who had received, twenty-three days previously, some shot in his face, from a gun, one of which had penetrated the eye, destroying it. When I arrived, his father said that his son had just fallen asleep a few minutes before, and asked if he should awaken him. I at once proposed to Drs. Light, Lineaweaver and Gilford, of that place, who were present, that we should not awaken him, but try to etherize him while asleep. We went up quietly into the room; Dr. Light took the towel, on which ether had been poured, and approached him gently and slowly, I renewing the ether from time to time, until the cloth was applied immediately over the face, and thus he was put completely under the influence of the anæsthetic without his waking up; he making only a slight roll over from one side to the other. He was then carried out into the adjoining room, in which the operation was to be made, laid on a settee arranged for that purpose, and the eyeball removed. He did not wake up from the time he went to sleep on his mother's bed, before I arrived, until after the operation was completely finished; and then he came to himself with the remark, "Let my eye alone."

Hoping that you may be the means of drawing this needed information together as a safe-guard for the future, I remain, yours truly.

